# Influence of Zn Addition on the Aging Precipitate Behavior and Mechanical Properties of Al-Cu-Li Alloy

**DOI:** 10.3390/ma17071562

**Published:** 2024-03-29

**Authors:** Meiqi Wang, Lizhen Yan, Xiwu Li, Yongan Zhang, Zhihui Li, Kai Wen, Hongwei Liu, Baiqing Xiong

**Affiliations:** 1 State Key Laboratory of Nonferrous Metals and Processes, China GRINM Group Co., Ltd., Beijing 100088, China; wangmq0906@163.com (M.W.); zhangyongan@grinm.com (Y.Z.); lzh@grinm.com (Z.L.); wenkai@grinm.com (K.W.); lhw@grinm.com (H.L.); xiongbq@grinm.com (B.X.); 2 GRIMAT Engineering Institute Co., Ltd., Beijing 101407, China; 3 General Research Institute for Nonferrous Metals, Beijing 100088, China

**Keywords:** Al-Cu-Li alloy, Zn addition, T_1_ precipitate, mechanical properties

## Abstract

In the present work, the effect of Zn on the aging precipitates and mechanical properties of Al-Cu-Li alloys was investigated by Vickers hardness, tensile tests, transmission electron microscopy (TEM) and differential scanning calorimetry (DSC). The results indicated that the addition of Zn reduced the activation energy of the T_1_ phase and makes it easier to precipitate. The activation energy of the T_1_ phase, which was 107.02 ± 1.8 KJ/mol, 94.33 ± 1.7 KJ/mol, 90.33 ± 1.7 KJ/mol and 90.28 ± 1.6 KJ/mol for 0Zn, 0.4Zn, 0.8Zn and 1.2Zn alloy, respectively. The area number density of the T_1_ precipitate ranged from 97.0 ± 4.4 pcs/μm^2^ to 118.2 ± 2.8 pcs/μm^2^ as the Zn content increased from 0 to 1.2 wt.%. Consequently, the addition of Zn promoted the precipitation of the T_1_ phase. Therefore, the peak hardness and tensile strength of the alloy also increased with the increase in the Zn content, and the hardness of the alloy with Zn content of 1.2 wt.% increased by 16.5 ± 1.4 HV; meanwhile, the ultimate tensile strength increased by 46.5 ± 2.5 MPa. Therefore, the area number density of precipitates increased and improved the strength of the Zn-containing alloy.

## 1. Introduction

Al-Cu-Li alloys have lower density, higher strength, higher specific stiffness and fatigue properties, thus becoming the material of choice for weight and stiffness critical structures in the aerospace industry [1,2,3]. Al-Cu-Li alloy is aging strengthening, and the mechanical properties of the alloy depend greatly on the fine precipitates formed during aging. The principal alloying elements of Al-Cu-Li alloys are Cu and Li, and the microalloying elements include Mg, Ag, Zn, rare earth elements, etc. The main strengthening precipitates of the alloy are T_1_(Al_2_CuLi) phase, θ′(Al_2_Cu) phase, δ′(Al_3_Li) phase, β′(Al_3_Zr) phase and S′/S(Al_2_CuMg) phase [1,4,5,6].

The T_1_ phase is one of the most effective strengthening precipitates of the Al-Cu-Li alloy. The crystal of T_1_ phase is hexagon and it has an orientation relationship of [0001]T_1_//[111]_Al_, [1010]T_1_//[110]_Al_ [7]. The precipitation can be promoted by introducing strain before artificial aging treatment [8] Numerous studies have demonstrated that the inclusion of Mg, Ag, Mn, Zn, and rare earth elements (RE) in Al-Cu-Li alloys can significantly enhance their mechanical properties [9,10]. Furthermore, the addition of small amounts of these microalloying elements can also facilitate the precipitation of the T_1_ phase [5,11,12]. Meanwhile, other studies have also demonstrated that methods to promote T_1_ precipitate include the introduction of pre-stretching to increase nucleation sites prior to artificial aging [13,14], or the addition of small amounts of the microalloying elements such as Mg, Ag, and Zn. Zn element has solid solution strengthening and aging strengthening effects. Zn is usually added with Mg composite, to promote the diffusion of Cu atoms, so that the Mg-Cu-vacancy clusters aggregate. The nucleation and precipitation of T_1_ phase are promoted, and the strength and toughness of the alloy are improved [15]. Jiangwei Sun et al. [16] found that the mechanical properties of the alloys gradually improved with the increase in Zn content at T4, T6 and T8 conditions, and the Zn element effectively promoted the precipitation of T_1_ phase. The samples subjected to T6 treatment (aged at 175 °C for 32 h) exhibited excellent mechanical properties, with a yield strength (YS) of 597 MPa, ultimate tensile strength (UTS) of 660 MPa and elongation (EL) of 7.6% [17]. Xiaohui Zhu et al. [18] found that under the condition of T6, Zn and Mg interacted and promoted the precipitation of the T_1_ phase, while under the condition of T8, the influence of Zn on the precipitation of enhanced phase was weakened. Grumbmann et al. [5] investigated the structure and composition of the precipitated phases at different aging times using STEM-HAADF coupled with EDS. The results showed that Zn was absorbed into the T_1_ phase, probably replacing the Cu atoms in the structure. Kertz et al. [19] also detected precipitation of T_1_ (Al_2_(Cu, Zn)Li) phases with different Zn contents on high angle grain boundaries by atomic probe microscopy and mass spectrometry. The results showed that Zn probably occupies Cu atoms in the T_1_ precipitate, and the addition of Zn did not significantly change the precipitation sequence.

According to all the studies mentioned, the simultaneous addition of Mg and Zn in the Al-Cu-Li alloy can effectively promote the precipitation of the T_1_ phase [5,9,10,11,12,13,14,15,16,17,18]. However, the Zn content that gives the optimum mechanical properties and its promoting mechanism in Al-Cu-Li alloy are still unknown. Consequently, the objective of this work is to investigate the effect of Zn content on the precipitates and the mechanical properties during aging process, and explore the mechanism of Zn element.

## 2. Materials and Methods

The raw materials of the alloys were industrial pure Al, pure Cu, pure Li, pure Zn, pure Mg, as well as Mg-30Zr and Al-10Mn intermediate alloys. First, the pure Al and Al-10Mn intermediate alloys were melted in a graphite crucible in a resistance furnace at 740 °C. Subsequently, pure Zn, pure Mg and Mg-30Zr intermediate alloys were added to the melt. After the alloy was melted, the melt was stirred with a graphite agitator [20]. The molten metal was degassed at 740 °C and C_2_Cl_6_ was introduced as the refining agent. The melt was then cooled to 720 °C and kept warm for 10 min to ensure that the alloying elements are completely homogenized; then, the molten metal was poured into a copper mold that was cooled using a water-cooling apparatus. 

Table 1 shows the chemical composition of the alloys. They were named 0Zn, 0.4Zn, 0.8Zn and 1.2Zn alloy. The as-cast ingot was processed with a two-step homogenization treatment, which was held at 390 °C for 16 h firstly, then held at 495 °C for 36 h, then quenched in air. The homogenization ingots with a diameter of 118 mm were extruded into a strip with a width of 62 mm and a thickness of 16 mm, with an extrusion ratio of 11.0. The extruded strips were immediately quenched in air after the extrusion process. Then, they were solution-treated at a temperature of 515 °C for 1 h, and aged at a temperature of 175 °C.

The age-hardening process was monitored by means of a Wilson VH1150 Vickers hardness tester (ITW TEST&MEASUREMENT (SHANGHAI) Co., Ltd., Shanghai, China) using a load of 5 kg and a dwell time of 15 s. The Vickers hardness of each sample was calculated by removing the maximum and minimum values from the seven test results and averaging the remaining five results. Tensile tests were conducted with CMT4304 testing machine (MTS SYSTEMS (CHINA) Co., Ltd., Shanghai, China) according to ISO 6892. The sample was φ5 × 60 mm, and tensile rate was 2.0 mm/min. All experimental results were the average of two parallel samples. A differential scanning calorimetry (DSC) model DSC 404F3 (NETZSCH, Shanghai, China) was used to test the melting temperature of the precipitated phases in the alloy samples, and an Al crucible was used in the process, heated in an Ar gas atmosphere at a rate of 5 K/min, 10 K/min, 15 K/min, 20 K/min and the test temperatures ranged from 20 °C to 600 °C. Peak temperatures (T_p_) were determined using the matching NETZSCH Proteus Thermal Analysis 8.0.3 analysis software. Transmission electron microscope (TEM) observation was conducted on a Talos F200X microscope (Thermo Fisher Scientific, Shanghai, China) operating at 200 kV. The sample was first mechanically thinned to 100 mm and then double-jet polished in a mixture of 75% CH_3_OH and 25% HNO_3_ in a temperature range of −25 °C and −35 °C. The number and diameter of the precipitated phase were calculated using Image Pro Plus 6.0.0., and 3 dark-field images of each alloy were calculated. Gatan Digital Micrograph 3.50 software was used to analyze the types of precipitated phases. 

## 3. Results

### 3.1. Artificial Age-Hardening Behavior

**Figure 1:**
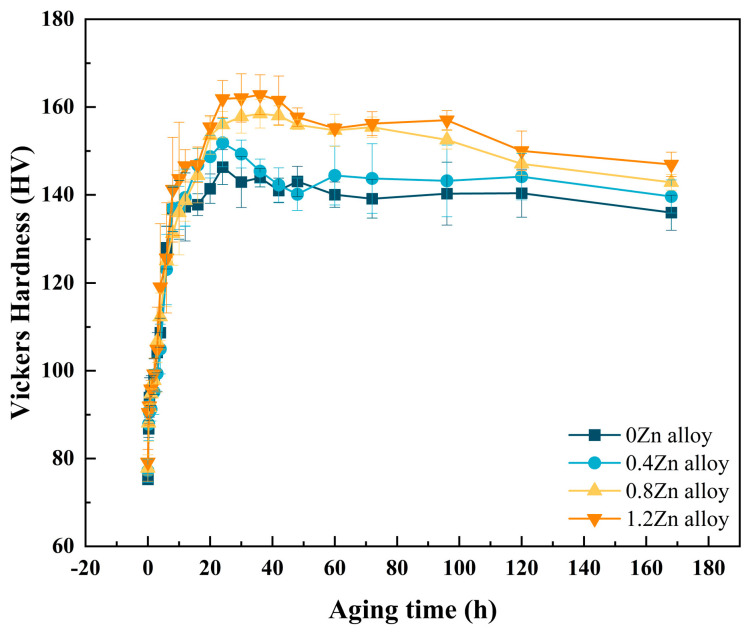
Vickers hardness vs. aging time curves for the alloys aged at 175 °C.

Figure 1 displays the Vickers hardness of the alloy aged at 175 °C as a function of aging time. All the alloys exhibited a strong age-hardening response. The hardness of the alloy increased rapidly within 12 h of aging at 175 °C; then, the growth rate slowed down and the alloy reached the peak hardness. After that, with the extension in the aging time, the hardness showed a decreasing trend. The peak hardness of the Zn-added alloys was increased compared to that of the Zn-free alloy, especially as Zn addition of 0.8 wt.% and 1.2 wt.%, which resulted in a substantial increase in hardness. The hardness of the alloy with a Zn content of 1.2 wt.% increased by 16.5 HV ± 1.4. The peak hardness values of 0Zn alloy, 0.4Zn alloy, 0.8Zn alloy and 1.2Zn alloy were 146.3 HV ± 3.1, 151.9 HV ± 3.0, 158.5 HV ± 2.6 and 162.8 HV ± 3.0, respectively, corresponding to peak aging regimes of 175 °C for 24 h, 175 °C for 24 h, 175 °C for 36 h and 175 °C for 36 h. As shown in References [8,19], the increase in Zn content in this investigation also delayed the peak aging state to some extent.

### 3.2. Mechanical Properties under Peak Aging Temper

**Figure 2:**
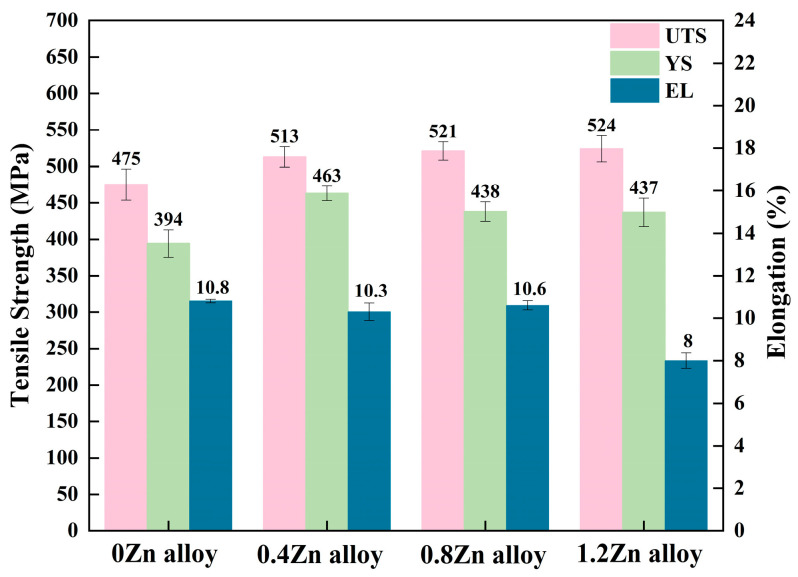
Mechanical properties of the alloys in peak-aged state.

Figure 2 shows the mechanical properties of the four alloys in the peak-aged condition. The ultimate tensile strength (UTS) of the alloys are 475 ± 21 MPa, 513 ± 19 MPa, 521 ± 17 MPa and 524 ± 25 MPa, for 0, 0.4, 0.8 and 1.2% Zn addition, respectively. As a consequence, the UTS increased with the enhancement of Zn content, while the strength of the alloys were basically equivalent when the additions of Zn were 0.8 wt.% and 1.2 wt.%. Compared with the 0Zn alloy, the UTS of 1.2Zn alloy is increased by 46.5 ± 2.5 MPa. The YS of the alloy is 394 ± 19 MPa, 463 ± 10 MPa, 438 ± 19 MPa and 437 ± 28 MPa, for 0, 0.4, 0.8 and 1.2% Zn addition, respectively. And the addition of Zn also improves the yield strength of the alloy. The relations between hardness and UTS and hardness and YS calculated by M. Tiryakioǧlu et al. [21] also indicated that the increase in Zn content increases the UTS and YS of the alloy. The elongations of the alloys were 10.8 ± 0.1%, 10.3 ± 0.4%, 10.6 ± 0.2% and 8.0 ± 0.4%, for the 0, 0.4, 0.8 and 1.2% Zn addition, respectively, which indicated that the Zn addition was less than 0.8 wt.% and had little effect on elongation; meanwhile, the elongation declined when the Zn content was more than 1.2 wt.%. 

### 3.3. Precipitation at the Peak-Aged Temper

**Figure 3:**
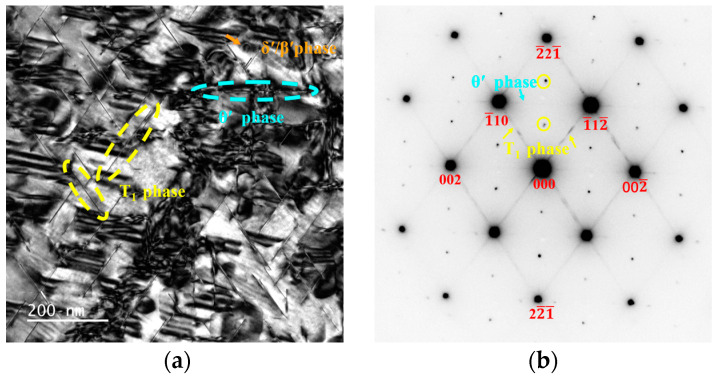

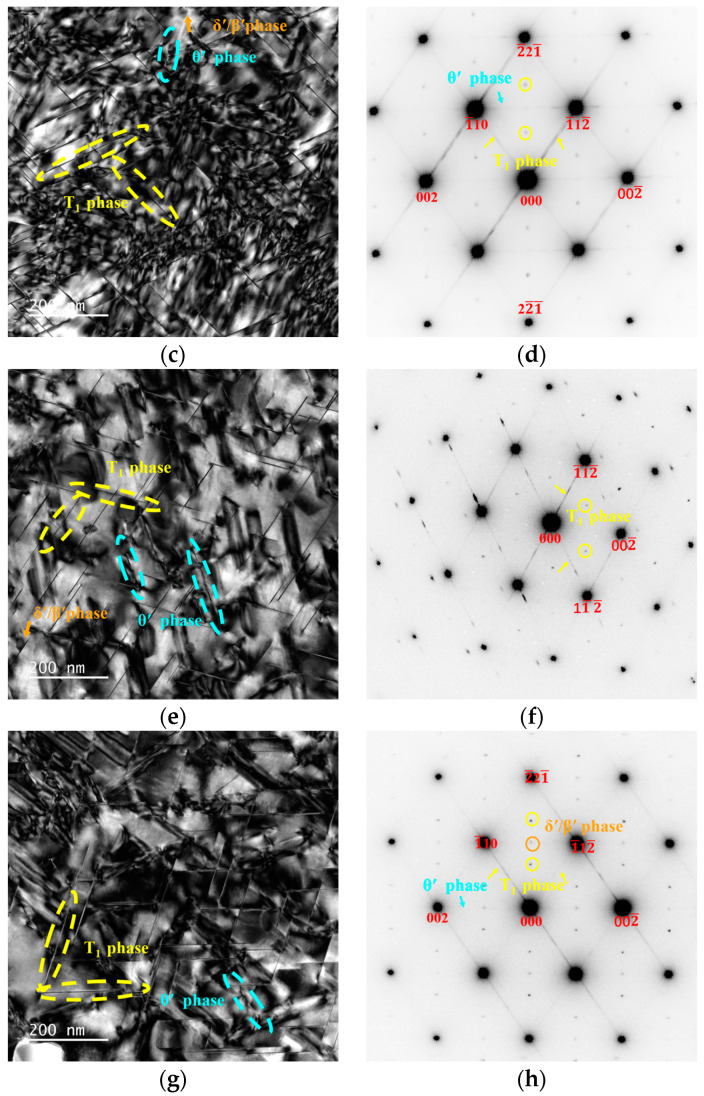
Bright field (BF) images (**a**,**c**,**e**,**g**) and selected area electron diffraction (SAED) patterns (**b**,**d**,**f**,**h**) taken along [110]_Al_ of the alloy: (**a**,**b**) 0Zn alloy; (**c**,**d**) 0.4Zn alloy; (**e**,**f**) 0.8Zn alloy; (**g**,**h**) 1.2Zn alloy.

Figure 3 shows the bright field (BF) image and selected area electron diffraction (SAED) patterns taken along the [110]_Al_ zone axis obtained from the 0Zn alloy, 0.4Zn alloy, 0.8Zn alloy and 1.2Zn alloy in peak aging temper. From the [110]_Al_ axis observation, T_1_ phase parallel to with to ($1\bar{1}\bar{1}$)_Al_ and ($\bar{1}1$$\bar{1}$)_Al_ can be observed. There is an approximate angle of 120° between them, namely the yellow dotted line in Figure 3 in the frames. The θ′(Al_2_Cu) phase is parallel to the ($00\bar{2}$)_Al_ phase within the blue box line in the figure [7,22]. T_1_(Al_2_CuLi) (yellow dotted line) and θ′(Al_2_Cu) (blue dotted line) were the main precipitates. The existence of some δ′/β′ precipitates can also be observed. Figure 3b,d,f,h display the SAED taken along the [110]_Al_ zone axis, where the precipitated phase diffraction spots shown in the bright field image can be observed. 

**Figure 4:**
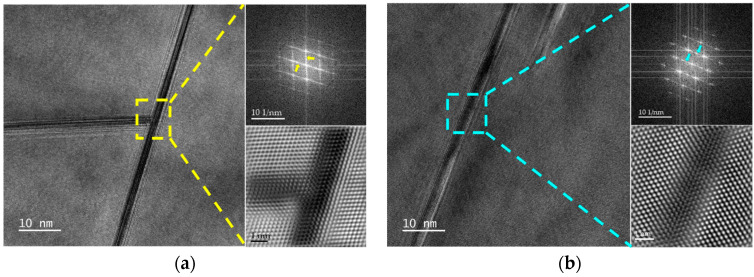
High-resolution transmission electron microscopy (HRTEM), SEAD and inverse Fourier variation patterns of the aging precipitate taken along the [110] zone axis: (**a**) T_1_ phase; (**b**) θ′ phase.

The representative high-resolution transmission electron microscopy (HRTEM) images of the alloy observed fom the [110]_Al_ zone axis and SAED as well as inverse Fourier transforms of selected regions are displayed in Figure 4. Viewing from the [110]_Al_ zone axis, two T_1_ phase variants in the ($1\bar{1}\bar{1}$)_Al_ and ($\bar{1}1$$\bar{1}$)_Al_ planes parallel to the electron beam can be seen [7,22]. Spots and diffraction patterns, as indicated by the yellow arrows in the diagram in Figure 4a in the upper right corner, also demonstrate the presence of T_1_ phases. When viewed from the [110]_Al_ zone axis, the phase parallel to ($00\bar{2}$)_Al_ is the θ′ phase. The spots and diffraction patterns in the diagram in Figure 4b in the upper right corner, which are indicated by the blue arrows, also indicate the θ′ phase.

**Figure 5:**
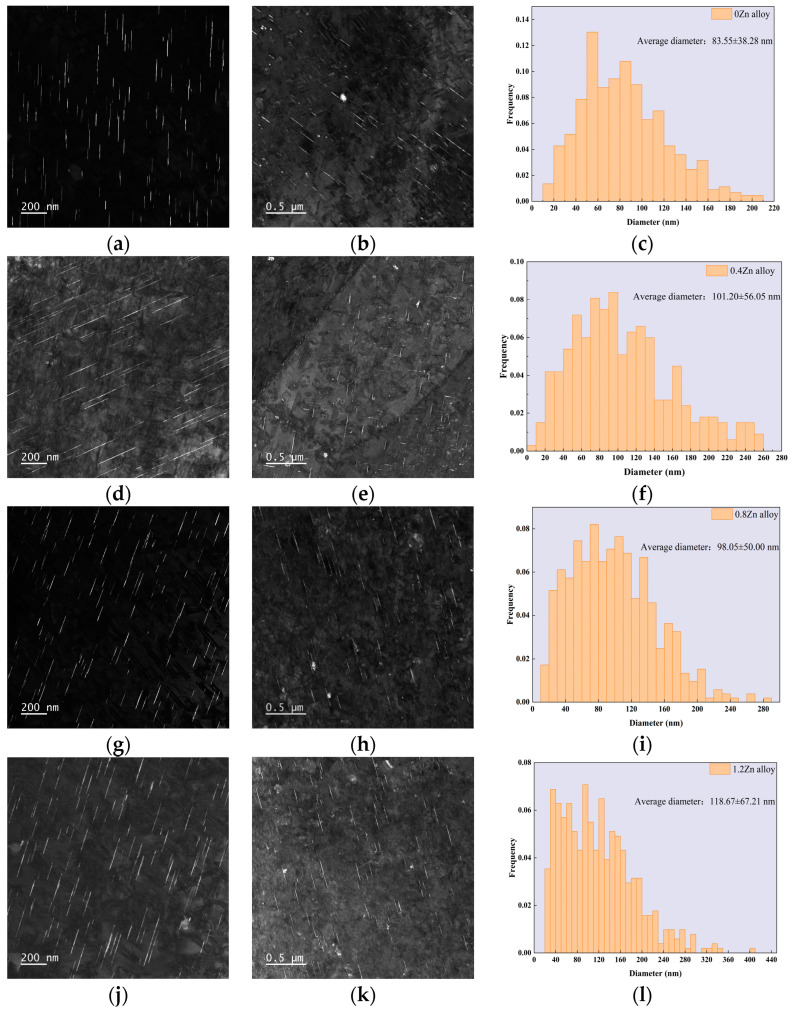
T_1_ phase dark-field (DF) images (**a**,**d**,**g**,**j**), θ′ phase and δ′/β′ phase dark-field images (**b**,**e**,**h**,**k**), and T_1_ phase diameter frequency distributions (**c**,**f**,**i**,**l**) of alloys in the peak-age state: (**a**–**c**) 0Zn alloy; (**d**–**f**) 0.4Zn alloy; (**g**–**i**) 0.8Zn alloy; (**j**–**l**) 1.2Zn alloy.

Figure 5a,d,g,j exhibit dark-field (DF) images of the T_1_ precipitate of the four alloys under peak aging state. It can been seen that the number of T_1_ phases in the alloys increased significantly with the addition of Zn. The number of T_1_ phases was counted as shown in Table 2. The area number density of T_1_ phases increased gradually with the enhancement of Zn content. The area number density of the T_1_ precipitates was 97.0 ± 3.1 pcs/μm^2^ for the 0 Zn alloy and 101.6 ± 2.4 pcs/μm^2^ for the 0.4 Zn alloy, while 114.8 ± 2.1 pcs/μm^2^ for the 0.8 Zn alloy and 115.6 ± 3.9 pcs/μm^2^ for the 1.2 Zn alloy. Figure 5b,e,h,k show the dark-field images of the θ′ precipitates for the four alloys, the small number of θ′ precipitates can be observed for the alloys and there is no significant difference. 

The addition of Zn in Al-Cu-Li alloy will reduce the solubility of Li atom in the matrix [23]. As shown by Hirosawa et al. [24], there is a relatively strong interaction between Zn and Li atoms. Thus, the Zn-Li binding was easier to form and can provide the required Li atoms for the T_1_ phase precipitation [17]. The binding ability between Mg and Cu atoms was slightly stronger than that of Zn and Cu [25], when the Zn addition is low, Cu binds preferentially with Mg atom; with the increase in Zn content, the probability of Cu binding with the Zn atom increases continuously [26], and due to the strong binding of the Zn and Li atoms, T_1_ phase precursors containing Zn, Li and Cu atoms and vacancies can be formed.

Figure 5c,f,i,l show the histograms of the frequency distribution of the diameters of the T_1_ phases of the 0Zn alloy, 0.4Zn alloy, 0.8Zn alloy and 1.2Zn alloy. It can be seen that the four alloys have a wide range of T_1_ precipitate size distributions and the size distribution range increases gradually with the rise in Zn content. The average diameters of the T_1_ phases also differed (as shown in Table 2), which were 83.55 ± 4.4 nm, 101.20 ± 5.0 nm, 98.05 ± 5.7 nm and 118.47 ± 2.8 nm of T_1_ phases for 0Zn alloy, 0.4Zn alloy, 0.8Zn alloy and 1.2Zn alloy, respectively. These indicated that the size of T_1_ precipitates increased with Zn addition, compared to the alloy without Zn addition.

The T_1_ precipitate was an important aging precipitation phase in the Al-Cu-Li alloy and was related to the strength and toughness of the alloys. With the addition of Zn element, the number of T_1_ precipitates increased, corresponding to the peak hardness and the strength of the alloy. The amount of Zn was more than 0.8 wt.%, the number of T_1_ precipitate in the alloy was basically equivalent, which is consistent with the mechanical properties of the alloy.

## 4. Discussion

### 4.1. Effect of Zn on the Age-Hardening Precipitates

From the kinetic point of view, the precipitation activation energy of the T_1_ precipitate in the alloy is calculated to further analyze the effect of the Zn element on the aging precipitation behavior of the alloy. The main methods currently used to calculate activation energy are the Ozawa [27], Boswell [28] and Kissinger [29] methods. In this investigation, the most commonly used and highly accurate Kissinger’s method is used to estimate the relationship between the peak top temperature $T_{p}$ and the heating rate V, and the Kissinger method is shown in Equation (1) [30,31]:

(1) $$\ln\left(\frac{{T_{p}}^{2}}{V}\right)=\frac{E_{a}}{RT_{p}}+C$$

where $T_{p}$ is the peak temperature of the precipitated phase at one heating rate; V is the heating rate; $E_{a}$ is the activation energy; R is the gas constant.

**Figure 6:**
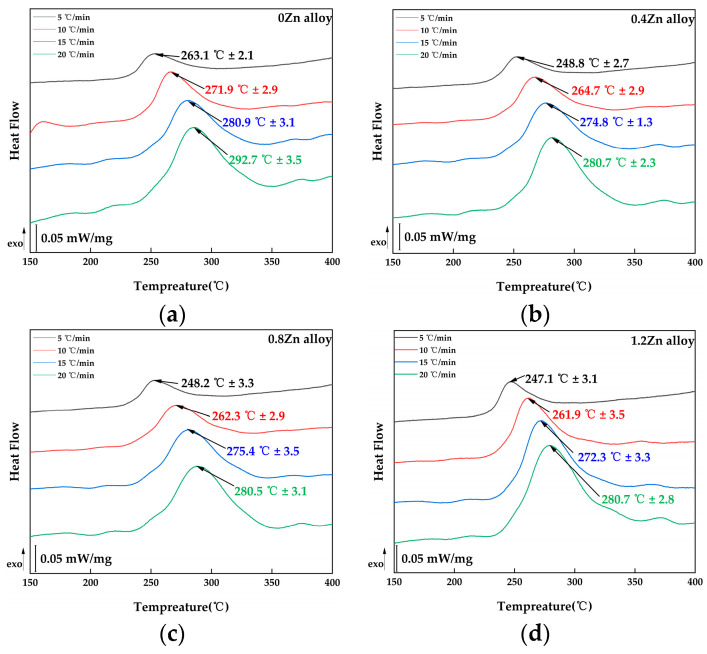
DSC results of solid solution alloys with different heating rates: (**a**) 0Zn alloy; (**b**) 0.4Zn alloy; (**c**) 0.8Zn alloy; (**d**) 1.2Zn alloy.

According to Kissinger’s method, the DSC curves of the alloys in solid solution are tested first and the results are shown in Figure 6. The heating rates used in the tests are 5 K/min, 10 K/min, 15 K/min and 20 K/min. From Figure 6, it can be seen that the precipitation temperature of the T_1_ precipitate on the DSC curve is shifted towards higher temperatures as the rate of heating increases, indicating that the precipitation of this phase is kinetically related.

**Figure 7:**
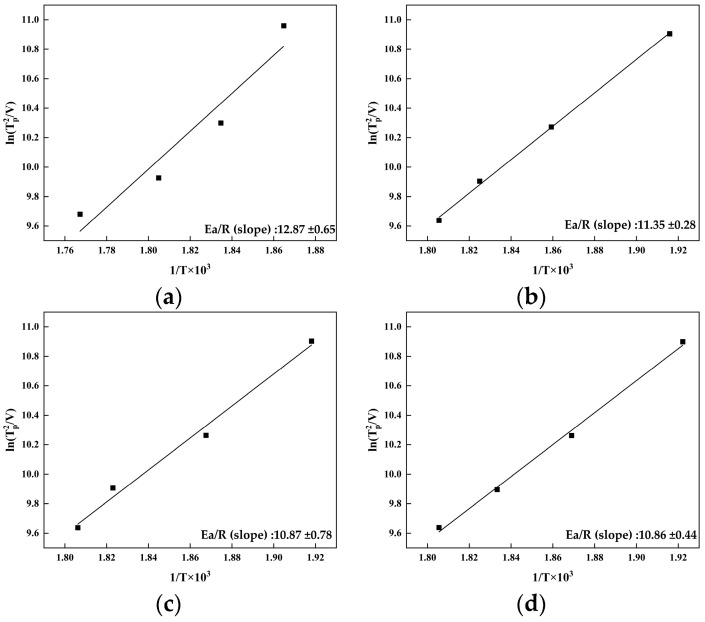
$\ln\left(\frac{{T_{p}}^{2}}{V}\right)$ − $\frac{1}{T_{p}}\;$ relationship curves for four alloys: (**a**) 0Zn alloy; (**b**) 0.4Zn alloy; (**c**) 0.8Zn alloy; (**d**) 1.2Zn alloy.

According to Equation (1), a single fitting is performed on the curve of $\ln(\frac{{T_{p}}^{2}}{V})$ versus $\frac{1}{T_{p}}$. Here, $\frac{E_{a}}{R}$ represents the slope of the $\ln(\frac{{T_{p}}^{2}}{V})$ versus the $\frac{1}{T_{p}}$ relationship curve, with R being the gas constant. By calculating the slope of the relationship curve, the activation energy of the T_1_ phase in the alloy can be determined. The fitted curves for the four alloys are shown in Figure 7.

The activation energy of the T_1_ phase in the four alloys was calculated by formula (1). Specifically, for the 0Zn alloy, the activation energy was 107.02 ± 1.8 kJ/mol; for the 0.4Zn alloy, it was 94.33 ± 1.7 kJ/mol; for the 0.8Zn alloy, it was 90.33 ± 1.7 kJ/mol; and for the 1.2Zn alloy, it was 90.28 ± 1.6 kJ/mol. This indicated that the addition of Zn reduced the activation energy of the T_1_ phase in the Al-Cu-Li alloy, thereby promoting the precipitation of the T_1_ phase. Furthermore, the activation energy decreased with an increase in the Zn content. The activation energy was essentially comparable at a Zn content of 0.8 wt.% and 1.2 wt.%.

### 4.2. Effect of Zn on the Mechanical Properties

The T_1_ phase is widely recognized as the main cause of precipitation strengthening in the Al-Cu-Li alloy and effectively disperses coplanar slip [17,32,33]. In this investigation, the peak hardness and tensile strength of the Al-Cu-Li alloy were increased by the addition of the Zn element, and the strength of the alloys were similar for a Zn addition of 0.8 wt.% and 1.2 wt.%. Statistical analysis of the area number density (Table 2) and size (Figure 5c,f,i,l and Table 2) of the T_1_ phase in the peak-aging state alloy show that the addition of the Zn element promoted the precipitation of the T_1_ phase, and with the increase in Zn, the area number density of the T_1_ phase rose, which was the reason for the improvement in the alloy performance. The T_1_ phase area number densities in the alloys with a Zn addition of 0.8 wt.% and 1.2 wt.% were basically the same, which explains the similar mechanical properties of the two alloys. The activation energy of the alloys was calculated using Kissinger’s method, and the results displayed that the addition of Zn can reduce the activation energy of T_1_ phase precipitation, and the activation energy of T_1_ phase decreases with the increase in Zn addition, which was the reason for the differences in the T_1_ phase in different alloys. It was worth noting that the activation energies of the T_1_ phase were approximate at the addition of 0.8 wt.% and 1.2 wt.%, and the ultimate tensile strength of two alloys was almost the same, while the 0.8Zn alloy exhibited high elongation, which indicated that the content of Zn should less than 0.8 wt.%.

## 5. Conclusions

In this work, the microstructure and mechanical properties of an Al-3.6Cu-0.9Li alloy with different Zn content during artificial aging were investigated. The following conclusions can be drawn:

(1) The addition of the Zn element improved the hardness and tensile strength of the alloy, and both peak hardness and ultimate tensile strength of the alloys increased with the enhancement of Zn. The peak hardness of the 1.2Zn alloy increased by 16.5 ± 1.4 HV and the ultimate tensile strength by 46.5 ± 2.5 MPa compared to the Zn-free alloy. The ultimate tensile strength of the alloys was essentially the equivalent for Zn addition of 0.8 wt.% and 1.2 wt.%, but the elongation was reduced for the 1.2Zn alloy.

(2) The addition of the Zn element did not change the type of precipitates, which were still the T_1_(Al_2_CuLi), θ′(Al_2_Cu) and δ′(Al_3_Li). Zn facilitated the precipitation of the T_1_ phase in the aging process, but the promotional effect on the T_1_ phase was similar when the Zn content was more than 0.8 wt.%. Additionally, Zn can effectively enhance the mechanical properties of the alloy, while the improvement in mechanical performance was approximately the same when the Zn content was 0.8 wt.% and 1.2 wt.%.

(3) The addition of the Zn element reduced the activation energy of the T_1_ phase in the alloy. The activation energy of 0Zn alloy, 0.4Zn alloy, 0.8Zn alloy, and 1.2Zn alloy were 107.02 ± 1.8 KJ/mol, 94.33 ± 1.7 KJ/mol, 90.33 ± 1.7 KJ/mol and 90.28 ± 1.6 KJ/mol, respectively. Thus, Zn promoted the T_1_ precipitate and improved the area number densities of the T_1_ phase, which enhanced the tensile strength. 

## Figures and Tables

**Table 1:** Measured chemical compositions of the alloy (wt.%).

Alloy	Cu	Li	Mg	Zn	Mn	Zr	Fe	Al
0Zn	3.54	0.81	0.42	—	0.38	0.10	<0.01	Bal.
0.4Zn	3.62	0.96	0.40	0.42	0.41	0.12	0.01	Bal.
0.8Zn	3.63	0.86	0.40	0.79	0.39	0.12	<0.01	Bal.
1.2Zn	3.56	0.90	0.42	1.22	0.40	0.11	<0.01	Bal.

**Table 2:** Area number density and diameter of T_1_ phase for the peak-aged alloy.

Alloys	Area Number Density (pcs/μm^2^)	Diameter (nm)
0Zn	97.0 ± 3.1	83.55 ± 4.4
0.4Zn	101.6 ± 2.4	101.20 ± 5.0
0.8Zn	114.8 ± 2.1	98.05 ± 5.7
1.2Zn	115.6 ± 3.9	118.67 ± 2.8

## Data Availability

Relevant data have been shown in the paper.
